# Enhanced Antibacterial Activity of Silver Doped Titanium Dioxide-Chitosan Composites under Visible Light

**DOI:** 10.3390/ma11081403

**Published:** 2018-08-10

**Authors:** Jie Li, Bing Xie, Kai Xia, Yingchun Li, Jing Han, Chunmao Zhao

**Affiliations:** 1 School of Materials Science and Engineering, North University of China, Taiyuan 030051, China; xiebingnuc@sina.com (B.X.); xiakainuc@163.com (K.X.); liyingchun@nuc.edu.cn (Y.L.); zhaochunmaonuc@sina.com (C.Z.); 2 School of Mechatronic Engineering, North University of China, Taiyuan 030051, China; ajingcool@tom.com

**Keywords:** cross-linked chitosan, silver, titanium dioxide, photocatalytic antibacterial

## Abstract

Nano titanium dioxide (TiO_2_) with photocatalytic activity was firstly modified by diethanolamine, and it was then doped with broad spectrum antibacterial silver (Ag) by in situ method. Further, both Ag doped TiO_2_-chitosan (STC) and TiO_2_-chitosan (TC) composites were prepared by the inverse emulsion cross-linking reaction. The antibacterial activities of STC composites were studied and their antibacterial mechanisms under visible light were investigated. The results show that in situ doping and inverse emulsion method led to good dispersion of Ag and TiO_2_ nanoparticles on the cross-linked chitosan microsphere. The STC with regular particle size of 1–10 μm exhibited excellent antibacterial activity against *E. coli*, *P. aeruginosa* and *S. aureus* under visible light. It is believed that STC with particle size of 1–10 μm has large specific surface area to contact with bacterial cell wall. The increased antibacterial activity was attributed to the enhancement of both electron-hole separations at the surface of nano-TiO_2_ by the silver ions under the visible light, and the synergetic and sustained release of strong oxidizing hydroxyl radicals of nano-TiO_2_, together with silver ions against bacteria. Thus, STC composites have great potential applications as antibacterial agents in the water treatment field.

## 1. Introduction

Antibacterial compounds, including inorganic compounds and organic polymers, have been applied as the new functional material in the field of water purification. Among them, chitosan, because of its good antibacterial activity, strong absorbing, sustained-release, environmental friendliness, and biocompatibility, appears to be one of the most promising antibacterial agents [1,2]. When the pH is lower than its p*K*_a_ (6.5), the protonated amino groups (NH_3_^+^) at the C2 position in the glucose monomer of chitosan chains allow for the formation of a polycationic structure, which can interact with anionic compounds of bacteria [3]. The amino and hydroxyl groups on the macromolecular backbone make chitosan physically and chemically react with various compounds [4,5,6]. For example, chitosan can react with terephthalaldehyde, forming a cross-linked chitosan hydrogel to make core-shell wall of the microcapsules applied in drug delivery system. The microcapsule is composed of a cross-linked chitosan hydrogel shell and an oily core containing drug molecules [7,8].

The composites of chitosan with inorganic materials (such as titanium dioxide) [9,10] or metallic materials (such as gold and silver nanoparticles) [11,12] can offer specific properties, such as broad spectrum antibacterial activity [13] and photocatalytic activity [14]. For example, titania-chitosan composites showed a photocatalytic effect on its antibacterial performance [15]. The hydroxyl radicals produced by titania under UV light hindered the growth of bacteria [16]. However, TiO_2_ with a wide band-gap can only exert its antibacterial properties under UV light [17,18,19]. Many methods have been investigated to achieve the visible light active TiO_2_ photocatalyst. The methods followed made visible light activity by the doping of TiO_2_ with transition metals [20]; doping of TiO_2_ with non-metals [21]; and coupling of TiO_2_ with grapheme et al. [22]. It could extend the absorption property in the visible region.

Among them, transition metals, such as silver (Ag) nanoparticles (NPs) doped titanium dioxide nanomaterials can offer enhanced visible light photocatalysis [23,24,25]. Also, Ag has strong antibacterial activities against both Gram positive and Gram negative bacteria, such as *Staphylococcus aureus* and *Escherichia coli*, which are common pathogens in water [26,27]. Moreover, the high specific surface area of Ag NPs makes it easier to release silver ions (Ag^+^) and then kill the bacteria [28]. Therefore, it is expected that silver doped titanium dioxide-chitosan composites would obtain enhanced antibacterial activity.

However, agglomeration of nano TiO_2_ and Ag NPs during the traditional doping process results in larger particles and decreased photocatalytic antibacterial properties [29]. Thus, it is necessary to modify the surface of nano titanium dioxide before use. Meanwhile, the preparation of silver doped titanium dioxide-chitosan composites by the inverse emulsion cross-linking reaction is also crucial for optimizing the antibacterial activities.

In this study, the surface of nano TiO_2_ was modified using diethanolamine and doped with Ag NPs by in situ method. Our goal of the introducing biocompatible chitosan as a carrier was to make it swell and release the Ag-TiO_2_ NPs with synergistic antibacterial activity in the water. The effects of glutaraldehyde concentration and inverse emulsion cross-linking reaction time on the particle size of the products were studied. The antibacterial activity of the composites with different particle sizes was examined by plate colony-counting methods. Moreover, antibacterial properties of chitosan, titania-chitosan (TC), and Ag doped TiO_2_-chitosan (STC) composites were compared under both visible light and dark conditions. Furthermore, the possible antibacterial mechanisms of the STC composites were proposed. When compared to TC, enhanced antibacterial activity of STC makes it possible to be used as an antibacterial agent in the field of water treatment.

## 2. Materials and Methods

### 2.1. Materials

Chitosan polymer (deacetylation degree of 95%, viscosity is 100–200 mPa·s), titanium oxide (TiO_2_, particle size < 40 nm), Span-80 (Sorbitan Fatty Acid Ester), phosphate buffer saline (PBS), and nutrient Agar were purchased from Aladdin Industrial Corporation (Shanghai, China). Paraffin liquid, ether, and acetic acid glacial were obtained from Fangzheng chemical reagent factory (Tianjin, China). Glutaraldehyde (50%) was obtained from Damao chemical reagent factory (Tianjin, China). Silver nitrate was obtained from Tianjin Fengchuan Chemical Reagent Technologies Co., Ltd (Tianjin, China). Sodium borohydride was obtained from Tianjin Kemiou Chemical Reagent Co., Ltd (Tianjin, China). Gram positive bacteria *S. aureus* (*Staphylococcus aureus*, CGMCC 1.2465), Gram negative bacteria *E.* *coli* (*Escherichia coli*, CGMCC 1.3373), and *P. aeruginosa* (*Pseudomonas aeruginosa*, CGMCC 1.2620) were obtained from China General Microbiological Culture Collection Center (Beijing, China). All of the reagents were used as received without any further purification.

### 2.2. Surface Modification of Nano TiO_2_

There are a large number of active hydroxyl groups on the surface of nano TiO_2_, resulting in the easy agglomeration of nano-scale TiO_2_ in the solvent due to the strong interaction of hydrogen bonds. Therefore, the nano TiO_2_ surface was modified to reduce the interaction of molecules. Firstly, 2.5 g nano TiO_2_ was dispersed in 80 mL ethyl alcohol to obtain homogeneous suspension through mechanical stirring, 0.09 mL diethanolamine was added to the suspension, stirred at a constant speed for 10 min, and then ultrasonically dispersed for 20 min at room temperature. Finally, the mixture suspension was suction filtered and washed with ethyl alcohol. The resulting solids were dried for use.

### 2.3. Preparation of Ag Doped Titania-Chitosan (STC)

**Figure 1:**
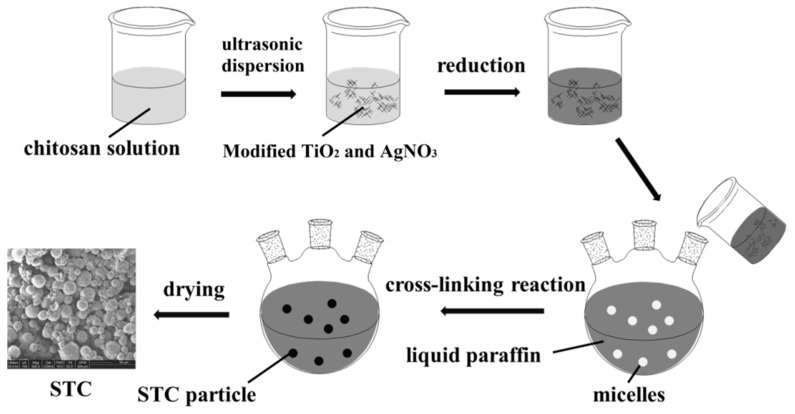
The fabrication process for Ag doped TiO_2_-chitosan (STC) composite.

Briefly, the 3 mL glacial acetic acid was added to the 197 mL deionized water to prepare 1.5 vol% glacial acetic acid solution. The chitosan solution was prepared by dissolving 0.3 g chitosan in 20 mL glacial acetic acid solution. It took one hour under ultrasonic dispersion at room temperature. Then, 0.1 g titania and 0.2 mL silver nitrate deionized water solution (1 mol/L) were added into chitosan stock solution. Next, this mixture was dispersed by ultrasonication for 30 min and the homogeneous suspension was obtained. After that, 0.4 mL sodium borohydride was added dropwise to the suspension and stirred for 30 min at room temperature, and then brown suspension was obtained. Finally, 200 mL liquid paraffin, certain amount (2, 3, 4, 5 mL) of glutaraldehyde, and 4 mL span-80 were added to the above suspension and stirred at 450 rpm for 2, 4, 6, or 8 h at 40 °C, the reaction was carried out in a nitrogen atmosphere. The mixture was filtered and the obtained solids were then washed three times with ether and distilled water, respectively, to remove the residual reagent. Final brown powders were obtained after vacuum drying the STC composites. Figure 1 illustrates the fabrication process for STC composite. Also, typical SEM image of prepared STC microsphere has been shown in Figure 1.

In order to investigate the photocatalytic antibacterial activity of STC composites, the titania-chitosan (TC) composites were prepared as well for comparison. The preparation process was the same, except that silver nitrate and sodium borohydride were not added.

### 2.4. Characterization

The molecular structure and interaction of chitosan, TC, and STC composites were investigated by Fourier transform infrared spectrometer (FTIR). The FTIR samples were prepared as KBr pellets, and the spectrum was collected at a resolution of 4 cm^−1^ with 32 scans per run by using a Bruker Tensor 27 FTIR spectrometer (Ettlingen, Germany). The morphology of the samples was characterized by using a JEOL JSM-6510 scanning electron microscope (Tokyo, Japan). The dried samples were coated with gold before the characterization for better imaging. The surface of the samples was analyzed by X-ray photoelectron spectroscopy (XPS) and Auger electron spectra (AES) in AXIS ULTRA DLD (Kratos Analytical Ltd., Manchester, UK) with Al Kα (1486.6 eV) radiation as the excitation source, the analysis was done in the pressures on the order of 10^−8^ Pa. The charges of the samples were corrected by setting the binding energy of the C_1s_ peak at 284.8 eV. The bandgap of the samples was characterized with UV/Vis spectroscopy (SHIMADZU UV-2550 spectrophotometer, Kyoto, Japan).

### 2.5. Antimicrobial Assays

The antibacterial activity of the samples (chitosan, TC and STC composites) was tested against three different bacterial species, i.e., two Gram negative bacteria, i.e., *E. coli* (*Escherichia coli*, CGMCC 1.3373) and *P. aeruginosa* (*Pseudomonas aeruginosa*, CGMCC 1.2620), and a Gram positive bacteria *S. aureus* (*Staphylococcus aureus*, CGMCC 1.2465). The ASTM E2180-07 (standard method for determining the antimicrobial effectiveness of agents that are incorporated into the polymeric surfaces) was adopted to evaluate the antibacterial activity of the samples. Briefly, the samples and all of the equipment and media were previously sterilized before antimicrobial testing. Firstly, a standard 0.5 McFarland suspension (1 × 10^8^ CFU/mL) was prepared. This suspension was further diluted to achieve a final test concentration of 1 × 10^4^ CFU/mL. Secondly, for each test, the samples (1 g) were made into 2 cm × 2 cm squares (the STC powder was slowly poured into the self-made 2 cm × 2 cm square hollow plastic mold, then pressed the powder into a sheet shape with square mass, and finally removed the mold to get the sample) and placed into a sterile Petri dish. Then, 0.5–1.0 mL inoculated agar slurry was pipetted onto the square test and control samples. The samples were placed in an incubator at 37 °C for 24 h under visible light and dark condition, respectively. Thirdly, the incubation samples were transferred to sterile beakers filled with 10 mL MH (Mueller-Hinton) broth. Then, the beakers were treated under ultrasonication for 2 min in order to facilitate the complete release of the agar slurry from the samples. At last, the diluted solutions were spread into nutrient agar and incubated at 37 °C for 48 h under the dark condition. After incubation, the bacteria colonies were counted and recorded by naked eyes. Visible light sources were supplied with 150 W (840 lm) halogen lamps (Philips, Shanghai, China). The distance between the light source and the sample is 20 cm. Each sample was performed in triplicate and tested, being repeated three times.

## 3. Results and Discussion

### 3.1. Structure and Morphology of STC Composites

**Figure 2:**
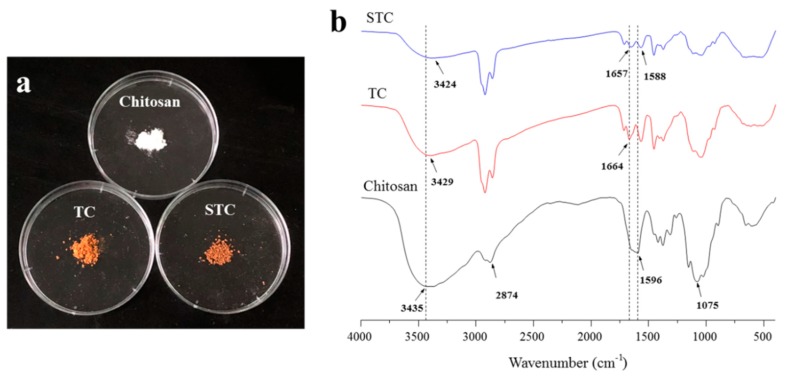
(**a**) Digital photos and (**b**) Fourier transform infrared spectrometer (FTIR) spectra of chitosan, TiO_2_-chitosan (TC), and STC.

Figure 2a shows the optical images of the chitosan, TC and STC composites. All of the materials were powder solid. Pure chitosan was white, whereas TC and STC composites were yellow, which might result from the cross-linking reaction between glutaraldehyde and chitosan in the context of the Schiff mechanism (The –NH_2_ of chitosan reacted with –CHO of glutaraldehyde to form C=N) due to the photochromic properties of Schiff base that led to the yellowing of TC and STC under illumination [30,31]. The molecular structure of the three samples was investigated by FTIR. In Figure 2b, for pure chitosan, the broadband located at 3435 cm^−1^ can be attributed to the hydrogenbonds of the O–H and N–H stretching vibrations, the peak at 2874 cm^−1^ corresponds to the vibration of C–H, the peak at 1596 cm^−1^ is related to the N–H deformation vibration, and the peak at 1075 cm^−1^ is due to the C–O stretching vibration [32]. When compared with pure chitosan, the absorption band of NH_2_ (1596 cm^−1^) groups for chitosan shifted slightly to a lower wavenumber (1588 cm^−1^) for TC and STC, as evidenced by the hydrogen bonding between the amino group of chitosan and the hydroxyl groups of TiO_2_ [31]. Moreover, the intensity of the superimposed peaks of hydroxyl and amino groups was significantly reduced in TC and STC, which could be attributed to the reduction of the primary amine groups by the reaction of the amino groups on the chitosan molecular chains with glutaraldehyde [33]. When compared with pure chitosan, the new bands at 1664  and 1657 cm^−1^ in TC and STC emerge, respectively, can be attributed to the formation of the C=N bond, further demonstrating the formation of the Schiff base by the reaction between chitosan and glutaraldehyde [31,33].

**Figure 3:**
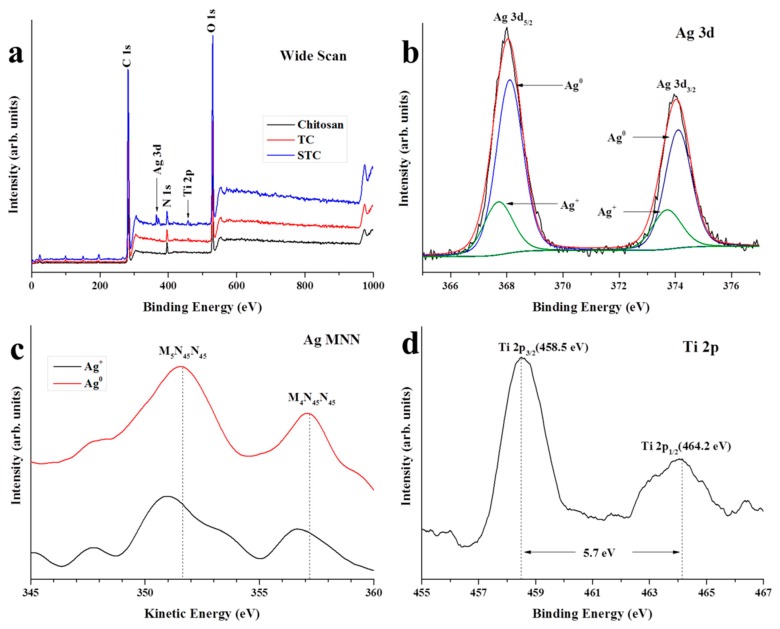
X-ray photoelectron spectroscopy (XPS) of chitosan, TC, and STC. (**a**) Wide scan; (**b**) High resolution XPS Ag 3d spectra on the surface of STC; (**c**) Ag MNN Auger electron spectra of STC; and, (**d**) High resolution XPS Ti 2p spectra on the surface of STC.

The X-ray photoelectron spectroscopy (XPS) was carried out to investigate the elementary composition on the surface of STC. In Figure 3a, for the wide scan spectra of the three specimens, the peaks at 285, 398 and 533 eV are corresponding to C 1 s, N 1 s, and O 1 s, respectively. The Ti 2p peak is found in the spectra of STC and TC (459 eV), and the Ag 3d peak is observed in the spectrum of STC (369 eV). It is demonstrated the presence of Ag and Ti elements on the surface of STC. The Ag 3d spectrum of STC composite was fitted with an asymmetric Gaussian-Lorentzian sum function while using the fitting program XPSPEAK41 (Figure 3b). It is clearly seen that the strong peaks at 368.1 and 374.1 eV are attributed to 3d_5/2_ and 3d_3/2_ of metallic silver (Ag^0^). While, the weak peaks at 367.7 and 373.7 eV are attributed to 3d_5/2_ and 3d_3/2_ of silver ion (Ag^+^) in silver oxides. It may be the result that a small part of Ag NPs on the surface of STC are oxidized during the storage process. The Ag 3d Auger analyses of STC composites were carried out to further confirm the above results (Figure 3c). Besides, the Ag MNN Auger curve agrees with that of the Ag^+^ in silver oxides with two peaks (M_5_N_45_N_45_ and M_4_N_45_N_45_) that are located around 350.9 and 356.6 eV, respectively [34]. On the other hand, two peaks appear at higher kinetic energies (351.7 and 357.3 eV) are assigned to metallic silver, this further proves that Ag^0^ and Ag^+^ exist simultaneously on the surface of the STC [34,35]. Figure 3d shows the high resolution XPS spectrum of Ti 2p. Two peaks that were observed at 458.5 and 464.2 eV are associated with the splitting of Ti 2p_3/2_ and Ti 2p_1/2_, and the doublet separation of the peaks is 5.7 eV, proving the existence of TiO_2_ on the surface of STC [36].

**Figure 4:**
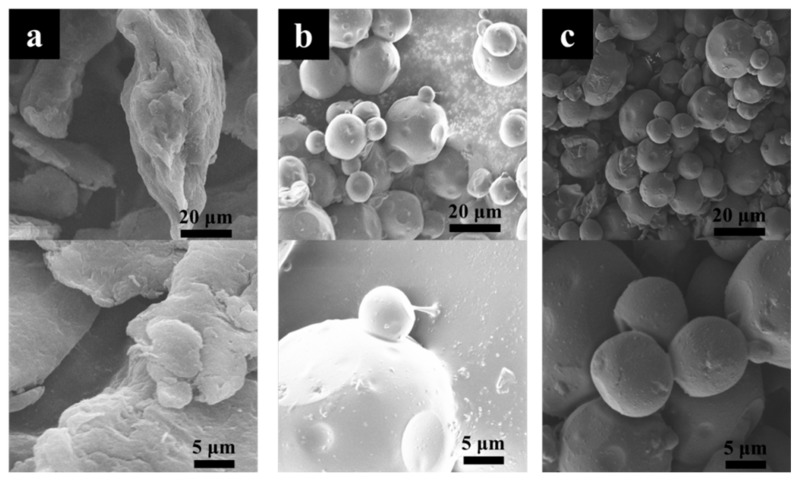
SEM images of (**a**) chitosan, (**b**) TC and (**c**) STC.

Figure 4 shows the microstructures of chitosan, TC, and STC composites. Chitosan presents an irregular sheet-like structure and dimensional inhomogeneity. After the reaction, similar morphologies were observed for the TC and STC composites. Both showed spherical micron particles, differing greatly from chitosan. It is probably because that with the addition of Span-80 surfactant during the preparation process, micelles were generated in paraffin, and then chitosan reacted with glutaraldehyde in the micelle structure, forming a spherical cross-linked product.

### 3.2. Effect of Particle Size on Antibacterial Activity

In order to study the influence of particle size on the antibacterial activity, a series of STC composite microspheres were prepared under different reaction parameters.

**Figure 5:**
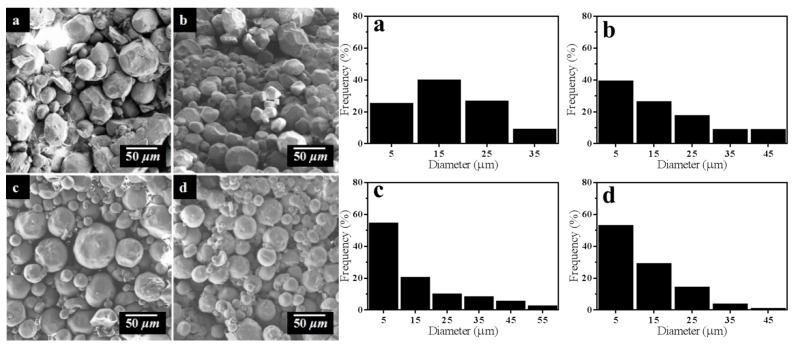
SEM images and particle size of STC composites with different glutaraldehyde dosage (**a**) 1.0%, (**b**) 1.5%, (**c**) 2.0%, and (**d**) 2.5% (*v*/*v*).

As we all know, the two aldehyde groups on glutaraldehyde molecule can react with the amino groups on two chitosan molecules, respectively, to make chitosan form a cross linked network structure. Thus, the influence of glutaraldehyde dosage on the morphology of STC is obvious. Four groups of samples with concentration of 1.0, 1.5, 2.0, and 2.5% (*v*/*v*) of glutaraldehyde were prepared and the results are shown in Figure 5. When the concentration of glutaraldehyde is 1.0% (*v*/*v*), the morphology of STC is extremely irregular. This is due to the concentration of being glutaraldehyde too low to have a full cross-linking reaction to form spherical particles. With increasing the concentration of glutaraldehyde to 2.0% (*v*/*v*), the particle morphology becomes more regular. When the concentration of glutaraldehyde increases continuously to 2.5% (*v*/*v*), the particle morphology is similar to the result of 2.0% (*v*/*v*), so it can be speculated that the 2.0% (*v*/*v*) concentration of glutaraldehyde can make the cross linking reaction become saturated.

**Figure 6:**
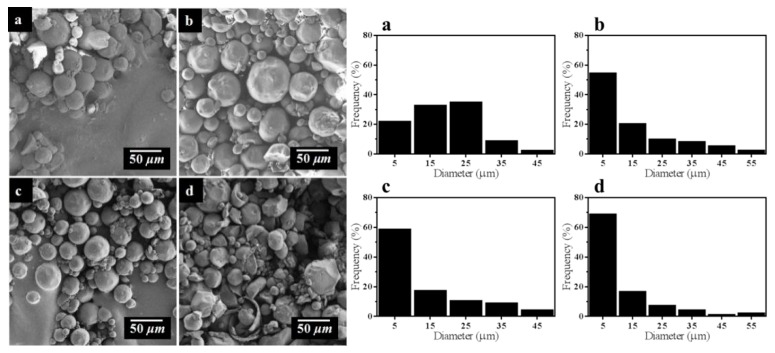
SEM images and particle size of STC composites with different cross-linking reaction time (**a**) 2 h, (**b**) 4 h, (**c**) 6 h, and (**d**) 8 h.

In addition, SEM images of four samples with inverse emulsion cross-linking reaction time of 2, 4, 6, and 8 h are shown in the Figure 6. The morphology of STC particles with 2-h reaction is irregular, i.e., a lot of non-spherical particles and wide particle diameter distribution. It is probably because that the cross linking reaction time is too short to form a complete sphere. When the reaction time is 4 h, the morphology of the particles is more regular. Furthermore, when the reaction time is 6 h, the morphology of the particles is the most regular in the samples. Nevertheless, when the reaction time is increased to 8 h, a part of particles are broken and the morphology is irregular. It is probably the result of the cross linked chitosan microspheres with large diameter being easily broken under shear stress and the long-time mutual collision between microspheres during the stirring process.

Further, size distribution of STC composites under different reaction parameters was summarized in Table 1. As shown in Table 1, with the increase of glutaraldehyde dosage, the quantity of STC particles with diameter between 1 and 10 μm increases from 25.00% to 54.34%. Besides, the particle diameter distribution of product becomes more concentrated and the regularity of particle is also improved. When the concentration of glutaraldehyde is 2.5% *v*/*v*, the particle size has no obvious change, which can be speculated that the optimal concentration of glutaraldehyde is 2.0% *v*/*v*. With increasing the reaction time, the percentage of STC particles with diameter between 1 and 10 μm increases from 21.74% to 68.75%, whereas the percentage of STC particles with diameter between 10 and 20 μm decreases from 32.61% to 16.67%, which means that the particle diameter distribution of product is more concentrated. However, the regularity of particle morphology is deteriorated when the reaction time is 8 h, thus it can be speculated that the optimal reaction time is between 4 and 6 h. The above results are agreed with SEM images of samples in Figure 5 and Figure 6.

**Figure 7:**
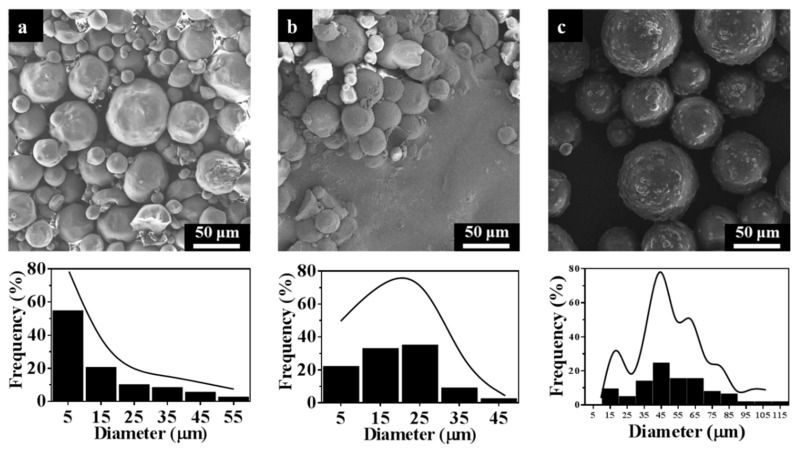
SEM images and particle size distribution of (**a**) STC-3, (**b**) STC-5, and (**c**) STC-8**.**

Based on the above analysis, STC-3, STC-5, and STC-8 with typical particle diameter ranges being chosen to study the effect of particle size on antibacterial activity. It can be seen from Figure 7 that the particle diameter ranges STC-3, STC-5 and STC-8 is centered in 1–10 μm, 20–30 μm, and 40–50 μm, respectively.

**Figure 8:**
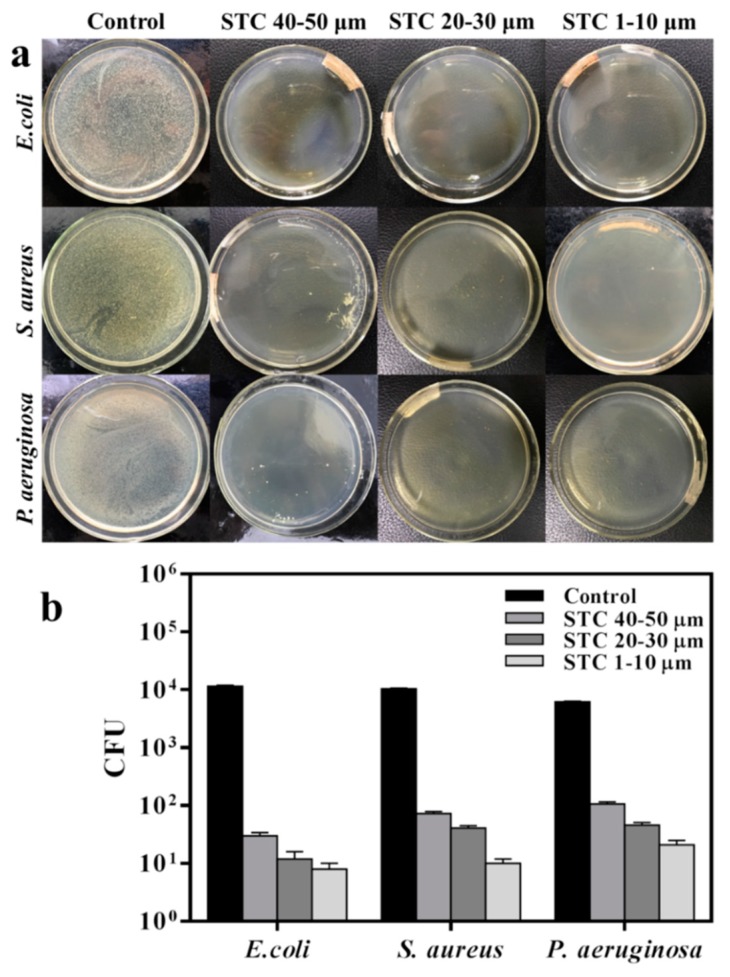
Antibacterial activities of STC-3, STC-5, and STC-8 with typical particle size. (**a**) Flat colony counting method (Standard Norm ASTM E2180–07) for STC composites with typical particle size (1–10 μm, 20–30 μm and 40–50 μm), against *E. coli* (CGMCC 1.3373), *S. aureus* (CGMCC 1.2465) and *P. aeruginosa* (CGMCC 1.2620); and, (**b**) Antibacterial test quantitative evaluation results. The agar plates (bacterial concentration of 1 × 10^4^ CFU/mL) without STC composites were treated as control group.

Next, the bacteria suspension with a concentration of 1 × 10^4^ Colony-Forming Units (CFU)/mL was inoculated on an agar plate as the reference group to compare the antibacterial abilities of STC-3, STC-5, and STC-8 with typical particle diameter ranges. All of the tests were conducted under the visible light condition and the results are shown in Figure 8 and Table 2. According to the bacterial colony picture (Figure 8a), it can be found visually that the STC-3 whose particle diameter are centered in 1–10 μm presents a best antibacterial property against *E. coli*, *S. aureus*, and *P. aeruginosa*. When the particle diameter of product are in the range of 20–30 and 40–50 μm, respectively, the number of bacterial colony increases successively as shown in Figure 8a and Table 2, which proves that when the particle diameter decreases, the number of bacterial colony decreases. This is probably because that the STC-3 with a smaller particle diameter has bigger specific surface area and higher surface energy [37]. Thus more STC particles can be in direct contact with the bacterial cells, so that silver ions and hydroxyl radicals, which work on the bacteria and then prevent the growth of bacterial, can be released from the silver and titania NPs stabilized on the surface of cross linked chitosan [38]. In addition, from the results that are shown in Figure 8b, the antimicrobial effect of STCs might be related to the species of bacteria. It can be found that the average colony-forming unit of *E. coli* (8 CFU) is the lowest among the three kinds, proving that the STCs have a best antibacterial ability on *E. coli*. Group of *P. aeruginosa* had the largest number of colonies (21 CFU), which indicated that the antibacterial activity of STC against *P. aeruginosa* was lower than that of *E. coli*. In addition, the colony-forming unit of *S. aureus* (10 CFU) was between that of *E. coli* and *P. aeruginosa*. Among the three bacteria strains, there were distinguishing characteristics in the compositions of their cell walls. Specifically speaking, the cell wall of Gram positive bacteria *S. aureus* is thicker and more rigid than that of Gram negative bacteria *E. colis*, which prevents the silver and titania NPs from entering bacterial cells [28]. For the *P. aeruginosa*, the bacterial biofilm will form a solid layer of envelope during the growth process of *P. aeruginosa*, which weakens the influence of STCs on the cell wall, this result is in good agreement with that already reported that the antimicrobial effect of antibacterial agent is related to bacterial species [28,39].

### 3.3. Effect of Visible Light Photocatalysis on Antibacterial Activity

**Figure 9:**
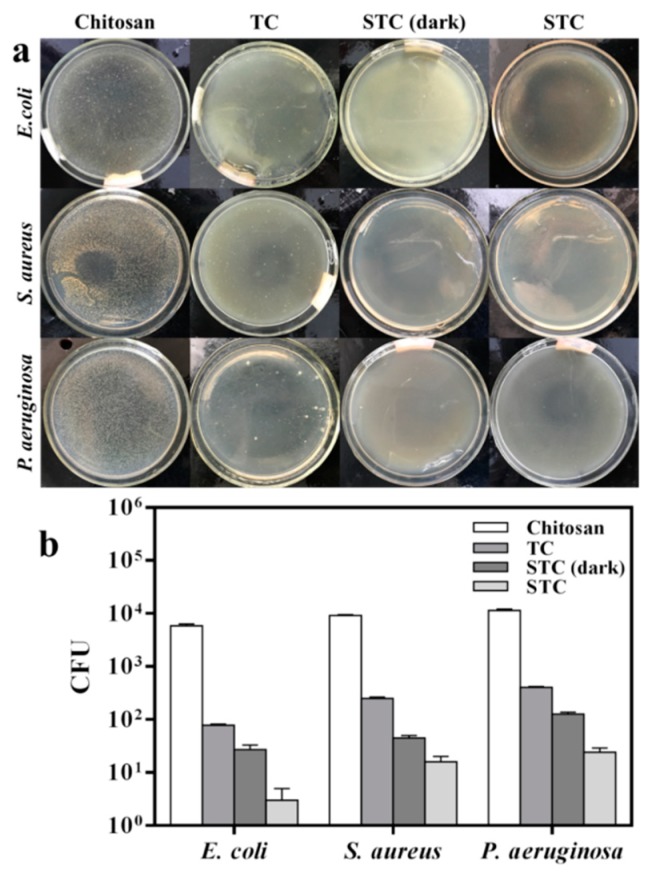
Effect of visible light photocatalysis on antibacterial activity. (**a**) Flat colony counting method (Standard Norm ASTM E2180–07) for chitosan, TC, STC-3 under visible-light conditions and STC-3 under dark conditions against *E. coli* (CGMCC 1.3373), *S. aureus* (CGMCC 1.2465), and *P. aeruginosa* (CGMCC 1.2620); and, (**b**) Antibacterial test quantitative evaluation results.

In order to study the photocatalytic antibacterial activity of STC, the antibacterial ability of STC-3 against several bacteria (Gram negative *E. coli* and *P. aeruginosa*, and Gram positive *S. aureus*) under visible-light and dark conditions was compared. In addition, tests of chitosan and TC composites under visible-light conditions were added. The results were denoted as colony-forming unit (CFU) and antibacterial rate presented in Figure 9 and Table 3. When compared with TC, the number of colonies in STC-3 treated Petri dishes is decreased significantly (Figure 9a). Especially under visible light irradiation, almost no colonies were found in all Petri dishes. This indicates that STC-3 exhibited the best antibacterial activity under visible light. As shown in Figure 9b and Table 3, it is clearly demonstrated that the STC-3 (1–10 μm) under visible-light and dark conditions both showed great antibacterial effect (AR > 98%) against all of bacteria strains, in comparison to chitosan and TC. Moreover, under visible-light conditions, the CFU of samples that are treated with STC was lower than that under dark conditions, indicating that the STC-3 showed stronger antibacterial properties under the visible-light irradiation.

### 3.4. Possible Antibacterial Mechanism

Based on the above analysis, the antibacterial mechanism of STC composites can be discussed, as below. On the one hand, the antibacterial activity of STC composites is related to the particle size. When compared with other STC composites with larger particle size, STC-3 with particle size of 1–10 μm has larger specific surface area, and its contact area with bacterial cells is greater. Therefore, STC-3 can directly act on the cell wall and inhibit the metabolism of bacterial. Furthermore, Liu et al. [40] have reported that silver ions can be released from the Ag NPs in the aqueous solutions under aerobic conditions, which could strongly interact with the thiol groups in the membrane proteins [38,41]. Further, the bacterial cell walls are destroyed, leading to bacterial death [38,41].

**Figure 10:**
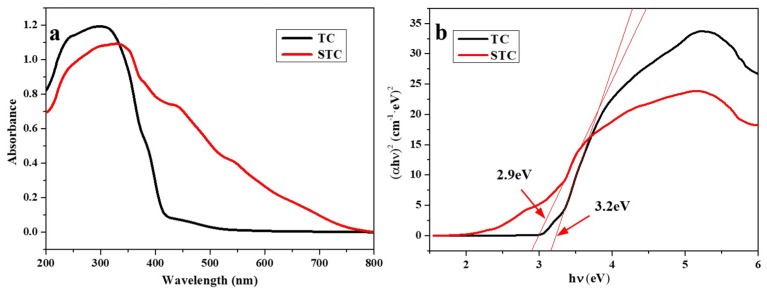
(**a**) Absorbance UV/vis spectra of TC and STC; and, (**b**) Determination of the band gap energy values for TC and STC.

On the other hand, the antibacterial activity of STC composites is also related to the photocatalysis process. To elucidate the underlying mechanism, the UV/Vis absorption spectra of TC and STC were characterized. FTIR spectra of the bacteria before and after the treatment with STC under/without light exposure were also recorded. As shown in Figure 10a, a strong absorption in UV range is observed for TC. This corresponds to the absorption peak of TiO_2_ in TC, indicating that TiO_2_ can only absorb ultraviolet light. Unlike that, the absorption spectrum of the STC composite contains the absorptions both in UV range and visible range (more than 430 nm). This indicates that the doping of Ag NPs to TiO_2_ can expand the photo response of TiO_2_ to the visible range. Due to the surface plasmon resonance of Ag NPs on the TiO_2_ is excited by visible light, the photocatalytic property of TiO_2_ is distinctly enhanced [42].

The method of UV/vis spectroscopy was employed to estimate band gap energies of the TC and STC. The absorption data were fitted to equations for indirect band gap transitions. The minimum wavelength that is required to promote an electron depends upon the band gap energy *E_bg_* of the photocatalyst and is given by Equation (1):

(1) $$\;E_{bg}=1240/\lambda \;\left(\mathrm{eV}\right)\;$$

where λ is the wavelength in nanometers [43,44].

Figure 10b shows the (α*E_bg_*)^2^ versus *E_bg_* for a indirect band gap transition of Ag NPs doped TiO_2_. Where α is the absorption coefficient and *E_bg_* is the photon energy [45]. It was found that the band gaps of TC and STC are 3.2 and 2.9 eV, respectively, indicating that the doping of Ag NPs to TiO_2_ can reduce the band gap of TiO_2_. The Ag NPs would inevitably affect the band structure of TiO_2_, and consequently affect the photo-activities of STC under visible light [42].

**Figure 11:**
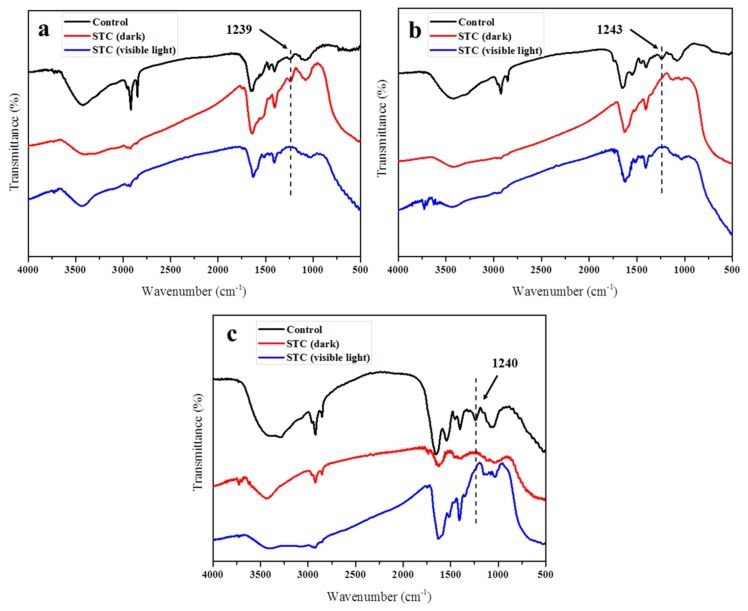
FTIR spectra of the (**a**) *E. coli* (CGMCC 1.3373), and (**b**) *S. aureus* (CGMCC 1.2465) and (**c**) *P. aeruginosa* (CGMCC 1.2620) before and after the treatment with STC under/without light exposure.

To confirm the damage of bacteria envelope, the FTIR of *E. coli*, *S. aureus* and *P. aeruginosa* was measured after STC treatment under/without light exposure. The results are shown in Figure 11. As we know, the bacterial membrane is made of phospho-lipids and protein [46]. For all bacteria, the peaks at 2840–2930 cm^−1^ and ~1240 cm^−1^ correspond to C-H bond and asymmetric vibration of PO_2_^−^ in phospho-lipids, respectively [47]. After being treated by STC in dark and visible light conditions (Figure 11a), the peak at 2840–2930 cm^−1^ significantly weakened, indicating that the group on cell membrane was damaged [46]. In particular, the absorption peak at ~1240 cm^−1^ disappeared after being treated by STC under visible light. The results showed that STC exhibited stronger antibacterial activity under visible light than in the dark condition. The antibacterial mechanism of the STC was confirmed by FTIR characterization.

**Figure 12:**
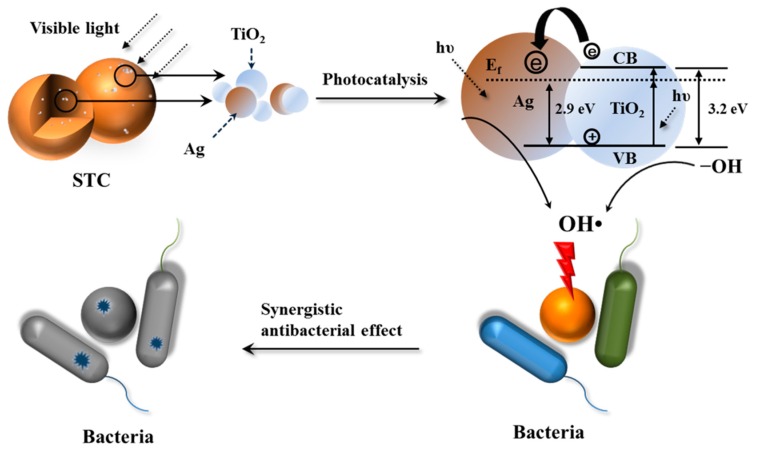
Antibacterial mechanisms of STC composites under visible light conditions. The first step: visible light radiation for STC. The second step: generation of active hydroxyl groups by photo initiation of Ag-TiO_2_. The third step: silver ions and active hydroxyl attack the bacterial cell wall. The fourth step: death of bacterial.

Based on the above results, the mechanism of photocatalytic activity of STC is proposed. Figure 12 shows the schematic diagram of antibacterial mechanism. Especially, under visible-light conditions, the surface plasmon resonance of Ag NPs on TiO_2_ is excited by visible-light irradiation [48]. Moreover, the recombination of the electron-hole pairs at the surface of TiO_2_ is inhibited by the silver ions acting as the electron traps, which are released from the Ag NPs doped in TiO_2_ in the aqueous solutions under aerobic conditions [40]. Then, silver ions were reduced to silver [49,50,51]. Thus, the surface electron excitation and electron-hole separation of TiO_2_ are enhanced [52]. Next, the photo induced holes oxidize the surface hydroxyl groups of TiO_2_. It leads to the release of hydroxyl radicals with strong oxidizing and antibacterial properties [23,24,49,52]. In addition, when the STC-3 is in the aqueous solution, the silver and TiO_2_ NPs that are incorporated in the cross-linked chitosan microspheres can be released slowly due to the swelling effects of cross-linked chitosan matrix, and the bactericidal performance can be sustained [53]. When compared to the traditional water disinfection technology (chlorine, ozone, and ultraviolet irradiation), STC is a better antibacterial agent [54].

## 4. Conclusions

The silver doped titania-chitosan (STC) composites were successfully prepared by the inverse emulsion cross-linking reaction. The antibacterial test results of the STC with different sizes demonstrated that reaction parameters are very important for the antibacterial properties of composites. Also, the antibacterial activity of STC against *E. coli*, *S. aureus*, and *P. aeruginosa* was improved with the decrease of particle size. The STC-3 with a regular particle size of 1–10 μm and good antibacterial activity was obtained, when the concentration of glutaraldehyde, reaction time and stirring rate were 2.0% (*v*/*v*), 6 h, and 450 r/min, respectively. In particular, STC-3 with particle size of 1–10 μm exhibited stronger antimicrobial activity under visible light than in dark conditions. For one thing, compared with other STC composites with larger particle size, STC-3 with particle size of 1–10 μm has larger specific surface area, and its contact area with bacterial cells is greater. Therefore, it can directly act on cell wall and inhibit the metabolism of bacterial. For another, when compared with TC, STC-3 with adding silver has enhanced antibacterial property, especially under visible light. Thus, in situ doping Ag in the modified nano-TiO_2_ was beneficial for the antibacterial activity enhancement of the samples under visible light, which was attributed to the enhancement of electron-hole separations at the surface of nano-TiO_2_ by the silver ions under the visible light, and the sustained release of strong oxidizing hydroxyl radicals of nano-TiO_2_ together with silver ions. This synergetic photocatalytic antibacterial activity makes them potentially applicable as antibacterial agents in the field of water treatment.

## 5. Patents

The method of silver doped titanium dioxide-chitosan composite antibacterial agent is protected by State Intellectual Property Office of the P. R. C (CN 105557753A).

## Figures and Tables

**Table 1:** Size distribution of STC composites under different reaction parameters.

No.	Reaction Parameters			Frequency of STC (1–10 μm) (%)	Frequency of STC (10–20 μm) (%)	Frequency of STC (20–30 μm) (%)	Frequency of STC (30–40 μm) (%)	Frequency of STC (40–50 μm) (%)
	Dosage of Glutaraldehyde (% *v*/*v*)	Reaction Time (h)	Stirring Rate (r/min)					
1	1.0	4	450	25.00	39.70	26.48	8.82	0
2	1.5	4	450	39.13	26.09	17.39	8.70	8.70
3	2.0	4	450	54.34	20.23	9.83	8.09	5.20
4	2.5	4	450	52.82	28.87	14.09	3.52	0.70
5	2.0	2	450	21.74	32.61	34.78	8.70	2.17
6	2.0	6	450	58.64	17.28	10.47	8.90	4.19
7	2.0	8	450	68.75	16.67	7.29	4.17	1.04
8	2.0	4	300	0	9.09	4.55	13.64	24.24

**Table 2:** The colony forming units (CFU) and antibacterial rate (AR) of STC with different size range.

Bacteria	Control	STC 40–50 μm	STC 20–30 μm	STC 1–10 μm
	CFU	CFU * (AR/%)	CFU * (AR/%)	CFU * (AR/%)
*E. coli*	1 × 10^4^	30 (99.70)	12 (99.88)	8 (99.92)
*S. aureus*	1 × 10^4^	73 (99.27)	41 (99.59)	10 (99.90)
*P. aeruginosa*	1 × 10^4^	107 (98.93)	46 (99.54)	21 (99.79)

* The mean number of the colony forming units in the three repeated experiments.

**Table 3:** The colony forming units (CFU) and antibacterial rate (AR) of each sample (Chitosan, TC and STC).

Bacteria	Control	Chitosan	TC	STC (Dark) *	STC
	CFU	CFU ** (AR/%)	CFU ** (AR/%)	CFU ** (AR/%)	CFU ** (AR/%)
*E. coli*	1 × 10^4^	5888 (41.12)	78 (99.22)	27 (99.73)	3 (99.97)
*S. aureus*	1 × 10^4^	9216 (7.84)	250 (97.50)	45 (99.55)	16 (99.84)
*P. aeruginosa*	1 × 10^4^	11456 (-)	406 (95.94)	127 (98.73)	24 (99.76)

* STC (dark) represents the tested sample in dark conditions. Other samples were tested under visible light conditions. ** The mean number of the colony forming units in the three repeated experiments.
